# Characterization of a (*pckA*) mutant of the non-nodulating bacterium *Rhizobium pusense* NRCPB10 induced by transposon *Tn5* mutagenesis

**DOI:** 10.1007/s13205-012-0057-5

**Published:** 2012-03-29

**Authors:** Subrata K. Das, Pran K. Chakrabartty

**Affiliations:** 1 Department of Biotechnology, Institute of Life Sciences, Nalco Square, Bhubaneswar, 751023 Orissa India; 2 Madhyamgram Experimental Farm, Bose Institute, Kolkata, 700129 West Bengal India

**Keywords:** *Rhizobium pusense*, *Tn5* transposon mutagenesis, *PckA*, Cloning

## Abstract

Phosphoenolpyruvate carboxykinase (PCK) catalyzes the decarboxylation and phosphorylation of oxaloacetate to phosphoenolpyruvate in the gluconeogenic pathway in most organisms. A *pckA* gene encoding PCK was cloned and sequenced from strain *Rhizobium pusense* NRCPB100, a spontaneous rifampicin resistant mutant of *R. pusense* NRCPB10^T^ (JCM16209^T^) of a recently described new species of a non-nodulating and non-tumorigenic bacterium. The mapping of the *pck* gene region following *Tn5* mutagenesis located the gene downstream of a transcriptional regulatory protein gene (c*hvI*) and upstream of a conserved hypothetical protein gene. The *pck* of 1,611 bp was deduced to encode 536 amino acids and showed high homology to the genes of known ATP-dependent PCK enzymes. Phylogenetic analysis of the gene placed it in a cluster with *pck* of other known members of Rhizobiales. Amino acid sequences of the putative functional regions of the deduced enzyme were found to be conserved.

## Introduction

Gluconeogenesis refers to the central metabolic pathway in which tricarboxylic acid (TCA) cycle intermediates such as oxaloacetate and malate are converted to hexose sugars to maintain hexose sugar homeostasis in cells. In many organisms, the first step of the gluconeogenic pathway is catalyzed by the enzyme phosphoenolpyruvate carboxykinase (PCK) (EC 4.1.1.49) which decarboxylates and simultaneously phosphorylates oxaloacetate to phosphoenolpyruvate (PEP) (Utter and Kolenbrander 1972). Fructose-1,6-bisphosphatase, which converts fructose-1,6-bisphosphate to fructose-6-phosphate, is required for gluconeogenesis (Østerås et al. 1997; Voet et al. 2008). PCK has also been demonstrated to play a crucial role in several metabolic processes associated with cataplerosis (Yang et al. 2009). PCKs have been classified according to their specificities for nucleotides, GTP and ATP. Generally, the enzymes from mammals and a number of other eukaryotes have specificity for GTP, while the enzymes from bacteria, yeast and plants have specificity for ATP (Utter and Kolenbrander 1972). In addition, all eukaryotes studied to date, also have copies of mitochondrial PCK (PCK-M) and cytosolic PCK (PCK-C) (Yang et al. 2009). PCK also appears to have an absolute requirement for divalent metal ions, such as Mn^2+^, but other divalent ions, such as Mg^2+^ or Co^2+^, can act as substitutes but with reduced activity (Utter and Kolenbrander 1972).

Besides the PCK reaction an alternative pathway exists in bacteria e.g. in *Escherichia coli,* in which oxaloacetate or malate is decarboxylated to pyruvate by oxaloacetate decarboxylase or malic enzyme, respectively (Hansen and Juni 1974). The pyruvate formed is then converted to PEP by PEP synthetase or by pyruvate: orthophosphate dikinase (Cooper and Kornberg 1967). Strains deficient in both oxaloacetate decarboxylase and malic enzyme cannot grow on dicarboxylic acid intermediates of the TCA cycle as sole carbon source.

Free-living *Rhizobium leguminosarum* bv. *viciae* (formerly *Rhizobium leguminosarum*) and *Ensifer meliloti* (formerly *R. meliloti,* then *Sinorhizobiummeliloti*) and *Rhizobium* sp. strain NGR234 require PCK, fructose-1,6-bisphosphate aldolase and fructose-1,6-bisphosphatase for growth and metabolism of C_4_-dicarboxylates through the gluconeogenic pathway (McKay et al. 1985, 1988; Finan et al. 1988; Østerås et al.^1991^). PCK deficient mutants of *Rhizobium* species fail to grow on minimal medium with succinate or other TCA cycle intermediates but are able to grow on glucose or glycerol as the sole carbon source suggesting that PCK functions in *Rhizobium* as a key gluconeogenic enzyme (Østerås et al. 1991). However, in *E. melilotipckA* appears to be regulated differentially in the bacteroids than in free-living cells. During symbiosis, the host plant supplies C_4_-dicarboxylates, succinate and malate, to the bacteroids within nodules. The low PCK activities in the symbiotic phenotypes of PCK mutants of *E. meliloti* suggest that the PCK pathway is not operative in N_2_-fixing bacteroids and that PCK may be important in the process of infection rather than in gluconeogenesis. However, the understanding of the role of *pckA* in the root nodule symbiosis is further complicated by the finding that *RhizobiumpckA* mutants are symbiotically defective in some host plants but are phenotypically a wild type on other hosts (Østerås et al. 1991). In *E. coli, pckA* expression is induced by gluconeogenic substrates and the expression is regulated by the growth phase of the culture and by cAMP (Goldie 1984). In *Agrobacterium pckA* is induced by acidic pH and is involved in the expression of *vir* genes (Liu et al. 2005). PCK is essential for growth of *Mycobacterium tuberculosis* on fatty acids and the organism relies on gluconeogenesis to establish and maintain infection (Merrero et al. 2010). Deletion of the *pckA* in *M. bovis* led to the reduction in the capacity of the bacterium to infect and survive in macrophages suggesting that PCK activity is important during establishment of infection (Liu et al. 2003). On the other hand, PCK-C mRNAs in human were found to be more abundant in non-tumor tissues than in the tumors invoking the gene for PCK-C to define as a new negative marker for colonic tumors (Bluin et al. 2010). It appears that analysis of *pckA* and gene products may yield new information on the requirements for infection and for survival of a non-infective species**.**

Recently, we have isolated *R. pusense* NRCPB10^T^(JCM16209^T^ = LMG25623^T^ = NCIMB 14639^T^) a new species from the rhizospheric soil of chickpea (*Cicer arietinum* L), a close phylogenetic relative of *R. radiobacter* (Panday et al. 2011), which was previously known as *A. tumefaciens* (Young et al. 2001). The present investigation was carried out in search for a possible reason for its non-infectivity. Previously PCK^−^ mutants of *R. leguminosarum* bv. *viciae* were reported to have no symbiotic phenotype (Finan et al. 1988) and *pckA* mutant of *A. tumefaciens* was also shown to be highly attenuated in tumor inducing ability. These observations prompted us to study the *pckA* gene of *R. pusense* NRCPB10 for its non-infectivity. Here we report the cloning, physical localization, nucleotide sequence and phylogeny of the *pckA* gene of *R. pusense* NRCPB100*.R. pusense* NRCPB100 is a spontaneous rifampicin resistant mutant of the strain NRCPB10.

## Materials and methods

### Bacterial strains, plasmids and media

The bacterial strains and plasmids used in this study are listed in Table 1. *R. pusense* NRCPB10 was grown on yeast mannitol (YM) agar medium (Vincent 1970) containing rifampicin (50 μg/ml) and a rifampicin resistant spontaneous mutant strain NRCB100 was isolated. NRCB100 was grown either in YM medium or in rhizobium minimal medium (RMM), pH 6.8, containing: (g/l): K_2_HPO_4_, 2; KH_2_PO_4_, 1.5; NaCl, 0.15; NH_4_Cl, 0.5; MgSO_4_·7H_2_O, 0.5; CaCl_2_.2H_2_O, 0.01 and glucose, 10 as the carbon source**.***E. coli* was grown in Luria broth (Miller 1972). The media were solidified with agar powder (Difco, USA) at 15 g/l when necessary.

**Table 1:** Bacterial strains and plasmid

Bacterial strain	Relevent phenotype	Reference
*E.coli* strains		
WA803	*Met*^−^, *Thi*^−^	(Wood 1996)
DH5α	F^−^ф80d, *Lac*Z ∆, M15 ∆, (*Lac* ZYA–*arg* F), U169, *deo*R, *rec*A1, *hsd* R17, (r_k_^−^, M_k_^+^) *Pho*A, *SUP* E44λ^−^, *thi*-1, *gyr*A96, *rel*A1	Bethesda Research Laboratories Inc
HB101	F^−^*hsdS*20(r_B_^−^ m_B_^−^) *recA*13 *ara*-14 *pro*A2 *Lac*Y1 *gal*K2 *rps*L20 *xyl*-*5 mtl*-1 *sup*E44λ^−^	Promega
*Rhizobium pusense*		
NRCPB10 (wild type)	Sm^r^, Nx^r^, Rif^s^, Neo^s^	(Panday et al. 2011)
NRCPB100	Spontaneous Rif^r^ mutant of NRCPB10	This work
NRC43 (mutant)	Suc^−^, Fum^−^, Mal^−^, Pru^−^, Rif^r^, Neo^r^	This work
Plasmids		
pGS9	p15A replicon, Cm^r^, Neo^r^	(Selvaraj and Iyer 1983)
pSUP104	Broad host range Cm^r^, Tc^r^ pACYC184 derivative	(Figurski and Helinski 1979)
pRK2013	Tra^+^,Km^+^,ColE1 replicon	(Priefer et al. 1985)
pBlueScriptKS^+^	Ap^r^, *Lac*Z′, T7 Phil10 promoter, f1 *ori*	Stratagene
kSD1	8.5 kb *Eco*R1 fragment of *pck*A::*Tn5* insert of strain NRC43 in pBlueScriptKS^+^	This work
kSD2	1.611 kb PCR amplified fragment containing *pck*A gene of strain NRCPB100 in pGEM-TEasy	This work
kSD3	1.851 kb fragment containing *pckA* gene in pSUP104	This work

*Met* methionine, *Thi* thiamine, *Suc* succinate, *Fum* fumarate, *Mal* malate, *Pru* pyruvate, *Sm* streptomycin, *Nx* nalidixic acid, *Rif* rifampicin, *Neo* neomycin, *Cm* chloramphenicol, *Tc* tetracycline, *Km* kanamycin, *Ap* ampicillin, ^*r*^ resistance, ^*s*^ sensitive

### *Tn5* mutagenesis

*R. pusense* NRCPB100 grown in YM for 2 days at 28 °C was mutagenised by mating with the donor *E. coli* WA803 (Wood 1996) harboring the suicide plasmid pGS9:: *Tn5* (Selvaraj and Iyer 1983) grown in Luria broth for 12 h at 37 °C as described (Gautam et al. 2007). Transconjugants were selected by plating dilutions of the bacteria on RMM containing glucose plus succinate as the carbon sources and supplemented with 2,3,5-triphenyl tetrazolium chloride (30 μg/ml), rifampicin (50 μg/ml) and neomycin (100 μg/ml). After incubating the plates for 3 days white colonies of mutants defective in the utilization of dicarboxylic acids were isolated (Ronson et al. 1981). The mutants failed to grow on succinate as the carbon source.

### Bacterial growth

Growth of *R. pusense* NRCPB100 and the mutant NRC43 was analyzed in RMM containing succinate (10 g/l) as the carbon source. Inocula were prepared by growing the bacteria in YM medium and washing with RMM. Growth of the bacteria in RMM was monitored by measuring the optical density at 600 nm in a SPECORD210 spectrophotometer, Analytik Jena, Germany.

### DNA manipulation, cloning and sequencing

DNA was isolated according to standard protocol (Meade et al. 1982). Routine manipulation of DNA, plasmid isolation, construction of recombinant plasmids, electrophoresis of DNA and transformation were carried out according to standard procedures (Sambrook and Russell, 2001). Digestions with restriction enzymes and DNA ligation were performed according to manufacturers’ instructions (Promega, Inc. and New England Biochemical, Beverly, Mass.). Southern blotting was performed as described (Southern 1975).

Sequencing of the *Eco*RI DNA fragment of NRC43 containing *Tn5,* which was cloned in pBlueScript vector, was performed with T3 and T7 primers by a commercial source (Microsynth GmbH, Switzerland) using an Applied Biosystems DNA Sequencer and a ABI PRISM Big Dye Terminator Cycle Sequencing kit (Applied Biosystems). Reaction products were analyzed with ABI PRISM 310 Genetic Analyzer (Applied Biosystem). After the first round, based on the partial sequence worked out new internal forward and reverse primers were designed and used to proceed with the next round of sequencing and the process was repeated each time with newly designed primers. Finally the chains of nucleotide sequences were assembled into a contiguous 2.737 kb sequence. ORFs were identified using the ORF finder facility of the National Center for Biotechnology Information (http://www.ncbi.nlm.nih.gov/gorf/gorf.html).

The oligonucleotide primers F1 (5′-ATGACCGAGATTGGGGTTCATAAT-3′) and R1 (5′-TTCTGCCGCGAGCAAAAGGCCCGG-3′) were designed from the sequences of the flanking regions of *pckA* gene of NRC 43 and were used for PCR amplification of 1.611 kb stretch of DNA, the complete coding region of *pckA* gene (ORF2), using *R. pusense* NRCPB100 genomic DNA as template. The resulting amplicon was purified and cloned into pGEM-TEasy plasmid vector (Promega, USA) for sequencing.

### Construction of plasmids for complementation

The oligonucleotide primers *pckA*F (5′-GCCGAATTCATTGTGGCAAAGGAAAAC-3′) and *pckA*R (5′-GATGAATTCTTCTGCCGCGAGCAAAAG-3′), designed from the DNA sequence of NRC 43 and were used for PCR amplification of about 1.851 kb of genomic DNA, which contained the *pckA* gene (ORF2) along with its promoter sequence, introducing the *Eco*RI site (underlined) into the PCR product. After digestion with *Eco*RI the amplicon was cloned into pSUP104 (Figurski and Helinski 1979) and *E. coli* HB101 was transformed with it to yield kSD3. The plasmid kSD3 was used to complement NRC43. Triparental spot mating was performed using the recipient NRC43, the donor kSD3 and the helper strain HB101/pRK2013 (Priefer et al. 1985). The ex-conjugants were identified by streaking on RMM containing glucose as the carbon source and supplemented with tetracycline and rifampicin.

### Genomic context analysis

Genomic context of *pckA* of *R. pusense* NCRPB10 in comparison with the genomes of related members of *Rhizobiales* was analysed using the GeConT programme. This programme is available at: http://www.ibt.unam.mx/biocomputo/gecont.html (Ciria et al. 2004).

### Phylogenetic analysis of the *pckA* gene

The *Eco*RI fragment of NRC43 containing *Tn5* was cloned in pBlueScript, sequenced, and a chain of 2.757 kb of nucleotides, which contained the *pckA* gene, was assembled. The ORFs in the sequence identified using BLASTX. BLAST searches provided the homologous sequences of the gene from the databases (Altschul et al. 1997). A neighbour-joining phylogenetic tree was constructed using *pckA* gene sequences of different organisms as per Kimura’s 2 parameter model (Kimura 1980) using MEGA software package, version 4.0 (Tamura et al. 2007). The tree topologies and statistical significance of branch points of the distance were tested by 1,000 bootstrap re-samplings of the data (Felsenstein 1985). The deduced amino acid sequences of the *pckA* genes were aligned using the ClustalW program package from EMBL (Heidelberg, Germany).

### Nucleotide sequence accession number

The nucleotide sequence data of *pckA* of *R. pusense* were deposited in GenBank database under accession number AF450091.

## Results and discussion

### Transposon mutagenesis and isolation of mutant

*Tn5* was introduced into *R. pusense* strain NRCPB100 using the suicide plasmid pGS9 containing *Tn5* from *E. coli* WA803 (Wood 1996). Transconjugants occurred at a frequency of 3.2 × 10^−4^ per donor. Transconjugants were selected on YM-agar plates containing neomycin and rifampicin. Over five thousand Neo^r^, Rif^r^ transconjugants were obtained. Screening on RMM containing glucose plus succinate as the carbon sources and 2,3,5-triphenyl tetrazolium chloride allowed the isolation of several white mutants of which NRC43 (Table 1) was arbitrarily chosen for further studies.

The mutant NRC43 failed to grow in RMM containing the glucogenic substrate succinate as was also shown for *pckA* mutants of *E. meliloti*, *R. leguminosarum* bv. *viciae* and *Rhizobium* sp. NGR234 (Finan et al. 1988; Østerås et al. 1991, 1995), whereas growth of the strain on mannitol or glucose as carbon source was similar to that of its parent. NRC43 also had growth, similar to its parent, on glucose, fructose, galactose, mannose, rhamnose, arabinose, xylose, lactose, maltose, mannitol or sorbitol as the carbon source but not on succinate, malate, fumarate, or pyruvate. The data indicated that the mutation in NRC43 did not allow it to grow in glucogenic substrates as the sole carbon and energy source, presumably the strain lacked the key enzyme of gluconeogenesis. On the other hand, growth of the parent NRCPB100 in the glucogenic substrates indicated that glucogenesis was operative in the strain, presumably due to induction of the key enzyme in the gluconeogenic pathway.

### Cloning and physical localization of the gene complementing NRC43

**Fig. 1:**
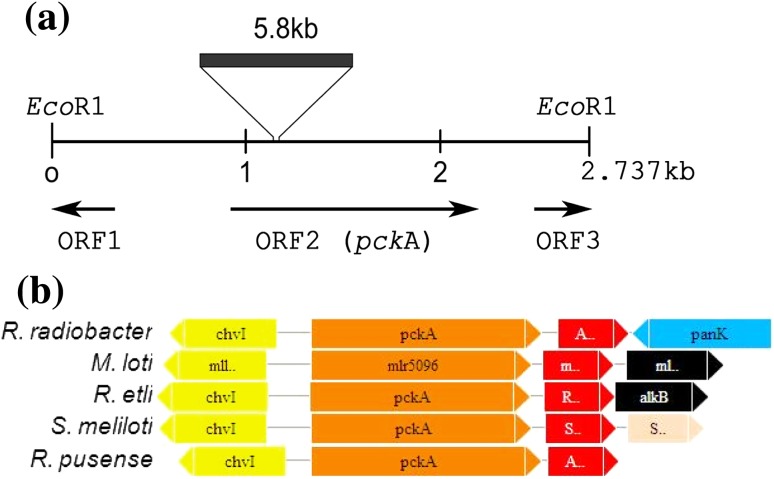
Genetic organization and genomic context of *Rhizobium pusense* NRC43 *pckA* DNA region. **a** Genetic organization of the *pckA* DNA region of *R. pusense* NRC43 and the transcriptional directions of ORF1, ORF2 (*pckA*) and ORF3 in the *Eco*RI digested DNA fragment containing *Tn5*. **b** Conservation of the genomic context of the *pckA* region in the genome of *R. pusense* in comparison with that of related members of Rhizobiale*s*. *Arrows* represent genes with their relative orientation in the genomes. Genes are colored according to their functional categories and labeled according to their original gene annotation in the database. *Yellow*, two component response regulator; *orange*, phosphoenol pyruvate carboxykinase; *red*, hypothetical protein; *blue*, pantothenate kinase; *black*, alkylated DNA repair protein; *light pink*, hypothetical protein

The *Eco*RI DNA fragment from *R. pusense* NRC43 containing *Tn5* was cloned into the pBluescript vector, transformed into *E. coli* strain DH5α and the recombinant plasmid kSD1 was isolated. The nucleotide sequence of the DNA flanking *Tn5* in kSD1 was determined using T3 and T7 primers. The sequence revealed one complete and two partial open reading frames (ORFs) (Fig. 1). Database sequence similarity searches showed that in the strain NRC43, *Tn5* had interrupted the gene homologous to *pckA* gene. The gene was flanked by two open reading frames; ORF1 upstream and ORF3 downstream of it. Analysis of the sequence of the 2.737 kb fragment also revealed that the ORF1 had the ATG start codon at position 851 which was preceded by a potential ribosome binding site, AGGAA, located 11 bp upstream. The putative transcription start site, AAAT, at position 730 was preceded by potential −10 (AAATAAAAT) and −35 (TTCAAC) sequences at positions 716 and 698 respectively, which were separated by 18 bp.

### Characterization of the *pckA* gene

The nucleotide sequence of ORF1 showed 91 % identity with the *chvI* gene sequence, however, the deduced protein product of the ORF showed 97 % homology with the transcriptional regulatory protein (ChvI) of *A. tumefaciens* C58. c*hvI* codes for a response regulator of a two-component regulatory system *chvGI* (Charles and Nester 1993). ORF2 was the *pckA* gene of 1,611 bp that encoded PCK consisting of 536 amino acids as was also found in *A.tumefaciens* (Liu et al. 2005). The deduced protein product shared 96, 81, 80, 78 and 77 % identity with that of *A. tumefaciens* C58, *R. etli* CFN42, *R. vitis* S4, *E. medicae* WSM419 and *E. meliloti* 1,021, respectively. The *pckA* is classified as an acid-inducible gene under the control of c*hvGI*, and is important in virulence of *A.tumefaciens* (Charles and Nester 1993). ORF3 was located downstream of the *pckA* gene and deduced protein product of the ORF shared 97 % homology with a conserved hypothetical protein of *A. tumefaciens* C58. All the open reading frames, except the ORF1, were transcribed in the same direction (Fig. 1a).

The genomic context of *pckA* gene in *R. pusense*, as compared to those in the related members of *Rhizobiales* showed that it was almost identical to that of *R. radiobacter*, although considerable homogeneity in composition with those of *E. meliloti*, *Mesorhizobium loti* and *R. etli* was also observed. All the four genomes were found to have gene orthologs for two component response regulator and phosphoenol pyruvate carboxykinase in their genome (Fig. 1b).

The *pckA* gene of 1,611 bp was amplified from *R. pusense* NRCPB100 by PCR using the primers designed from the flanking region of the ORF2 of NRC43. The amplicon was cloned into pGEM-TEasy vector, the recombinant plasmid kSD2 was isolated and the *pckA* in the clone was sequenced. The location of the *Tn5* insertion was determined from the sequence of the transposon insertion junction in the *pckA* gene. It was between nucleotides 436 and 438.

A stretch of 1.851 kb DNA from *R. pusense* NRCPB100 containing *pckA* and its promoter sequence was also amplified by PCR using primers designed from the flanking regions of the ORF2 of NRC43. The amplicon was cloned into the broad host range vector pSUP104 to yield kSD3. The promoter sequence was included for expression of *pckA* gene. Complementation of the mutant *R. pusense* NRC43 with the plasmid kSD3 resulted in a phenotype similar to that of its parent in respect of growth on succinate, malate, fumarate or pyruvate. The mutant NRC43 did not grow on glucogenic substrates such as C_4_-dicarboxylic acids as the sole carbon source. This suggested that an alternative pathway for synthesis of PEP (Hansen and Juni 1974) did not exist in *R. pusense* NRCPB100 as was also found in *A. tumefaciens* C58 (Liu et al. 2005). The results confirmed that in NRC43 *pckA* gene was biologically non-functional and complementation with *pckA* restored its function and gluconeogenic activity.

**Fig. 2:**
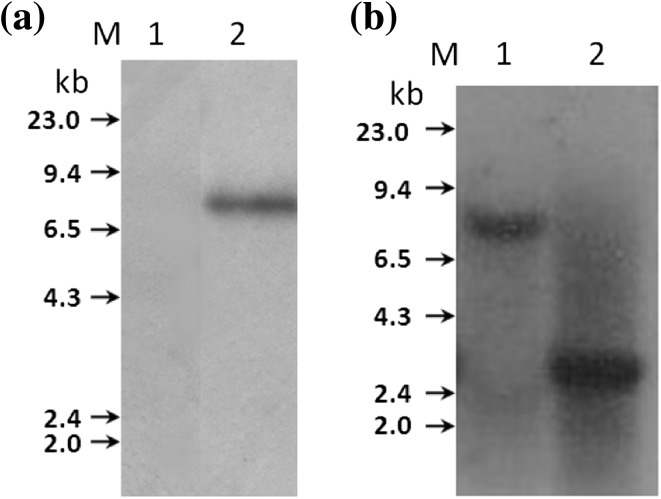
Southern hybridization analysis to localize the site of *Tn5* insertion in the strain NRC43 of *Rhizobium pusense* by hybridization using 1.2 kb DNA fragment containing neomycin gene as probe. **a** with *Eco*RI digested genomic DNA of NRCPB100 (*Lane 1*), and of NRC43 (*Lane 2*); **b***pckA* containing DNA sequence by hybridization of PCR amplified 1.611 kb fragment containing the *pckA* gene from the wild type NRCPB100 as a probe with *Eco*RI digested genomic DNA of strain NRC43 (*Lane 1*), and of strain NRCPB100 (*Lane 2*)

When genomic DNA from *R. pusense* NRC43 was digested with *Eco*RI and hybridized with ~1.2 kb ^32^P-labeled neomycin gene of *Tn5* as the probe a positive signal was obtained on a 8.5 kb DNA fragment of the strain NRC43 (Fig. 2a). To examine the location of *Tn5*, genomic DNA of NRCPB100 or NRC43 was digested with *Eco*RI and hybridized with the 1,611 bp *pckA* gene probe amplified by PCR from *R. pusense* NRCPB100 DNA. This resulted in the development of only one hybridization band signal on a *Eco*RI DNA fragment of about 8.5 kb of NRC43 or on a 2.737 kb *Eco*RI DNA fragment of NRCPB100 (Fig. 2b). The data demonstrated that there was only a single site of *Tn5* insertion in the chromosomal DNA of NRC43. Since *Tn5* does not have any *Eco*RI site on it, insertion of 5.8 kb *Tn5* in the 2.737 kb *Eco*RI DNA fragment of NRCPB100 increased its length to about 8.5 kb as observed in strain NRC43. From the data it was concluded that the phenotype of NRC43 was the result of insertion of *Tn5* in the 2.737 kb *Eco*RI DNA fragment which caused the inactivation of the *pckA* gene.

Mutation in the *pckA* gene exerts profound biological effects. PCK^−^ mutant of *R. leguminosarum* bv. *viciae* nodulated and fix N_2_ as effectively as its parent (McKay et al. 1985), while PCK^−^ mutants of *E. meliloti* showed reduced level of N_2_ fixation (Finan et al. 1991). The PCK enzyme, as such, was thought to be important in infection and nodulation. A PCK mutant of *Rhizobium* sp. strain NGR234 exhibited a host dependent symbiotic phenotype while N_2_ fixation by the mutant was also appreciably affected depending on host plants (Østerås et al. 1991). A *pckA* mutant of *A. tumefaciens* was attenuated in virulence (Liu et al. 2005) and *pckA*-deficient *M. bovis* BCG was also attenuated in virulence (Liu et al. 2003). The observations tempted us to analyze *pckA* gene of *R. pusense* NRCPB10 for its possible role in the strain’s non-infectivity. However, the *pckA* gene and the derivative enzyme of it were found to have high identity with those of other members of Rhizobiales which are infective. The enzyme of the strain was also metabolically active.

### Phylogenetic analysis

**Fig. 3:**
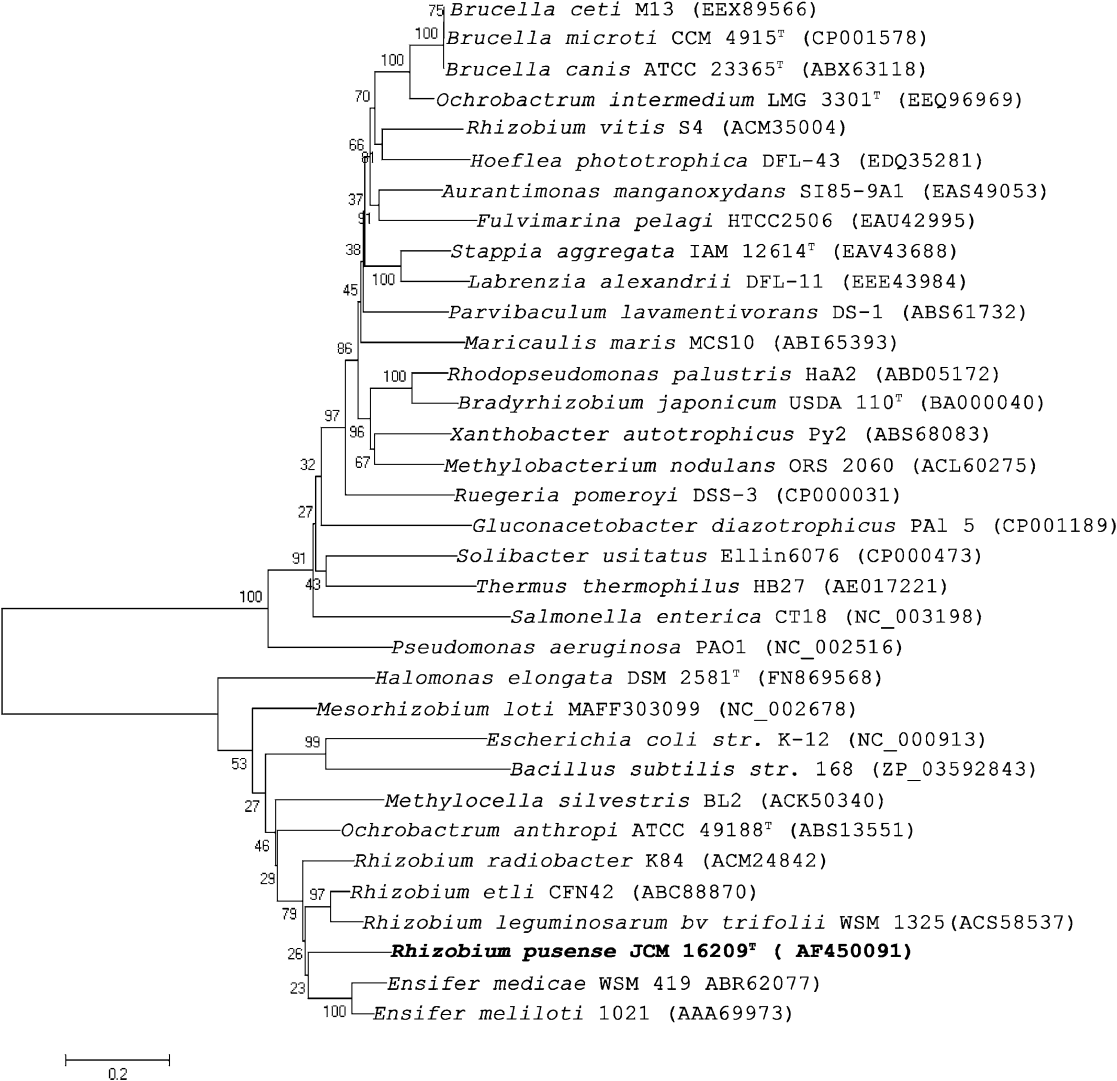
Neighbor joining tree showing the phylogenetic relationship of *Rhizobium pusense* (NRCPB100) with other related species based on 1,611 nucleotide bp of *pckA* gene sequence. Sequences showing similarity of more than 70 % only were considered in the phylogenetic tree. Bootstrap values based on 1,000 re-samplings are shown at the branch points. *Bar*, 2 nucleotide substitutions per 100 nucleotide position

**Fig. 4:**
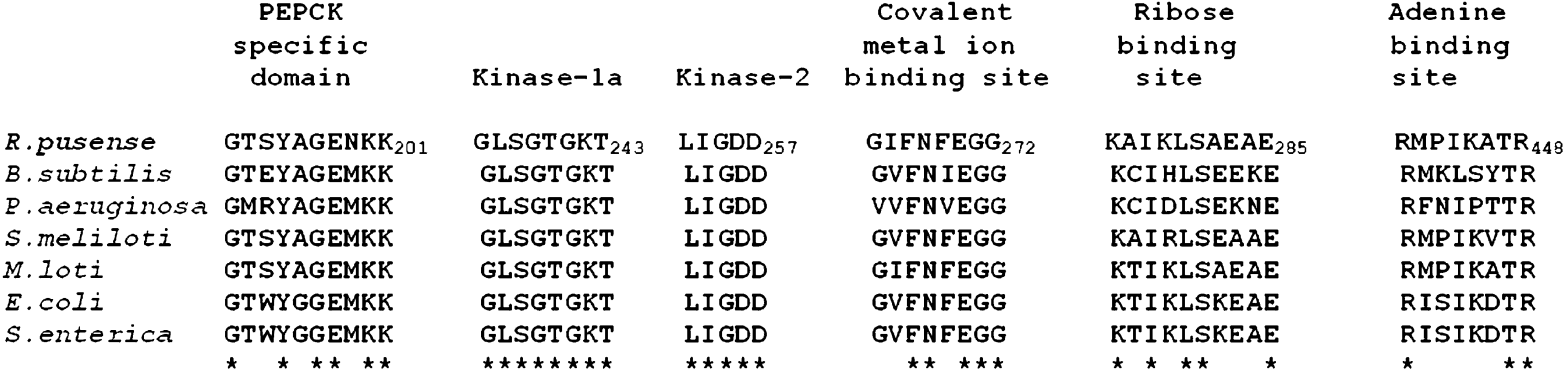
Conservation of the active site residues of ATP-dependent PCKs. Identical residues are indicated with *asterisks* below the sequence alignment. *Rhizobium pusense* (accession no: AAN86775); *Bacillus subtilis* (accession no: NP_390934); *Pseudomonas aeruginosa* (accession no: NP_253879); *Sinorhizobium meliloti* (accession no: NP_384151); *Mesorhizobium loti* (accession no: NP_105818); *Escherichia coli* (accession no: NP_417862) and *Salmonella enterica* (accession no: NP_458404). In the sequence of PCK of *R. pusense* the residue numbers are indicated immediately *right* to the residues

Phylogenetic analysis based on *pckA* gene sequence of *R. pusense* placed it in a cluster with *pckA* genes of other members of Rhizobiales (Fig. 3). Amino acid sequence alignment of the deduced enzyme showed that all of the putative functional regions were conserved in the ATP dependent PCK enzymes (Fig. 4). The PCK specific domain which is conserved in all ATP dependent PCKs analyzed to date was also found to be conserved in *R. pusense* enzyme (residues 192–201). In addition, kinase-1a (residues 236–243), kinase-2 (residues 253–257), the covalent metal ion binding site (residues 265–272) and ribose binding site (residues 276–285) were essentially identical among all the PCKs. On the other hand, more variability was observed in the adenine binding site, although the key residues, arginine and threonine (R_441_ and T_447_ in *R. pusense* NRCPB10), were identical to those of the others.

## Conclusion

A *pckA* mutant NRC43 of the recently described non-nodulating and non-tumorigenic species *R. pusense* NRCPB10 failed to grow on succinate. The *pckA* gene of 1,611 bp encoding PCK was located between the transcriptional regulatory protein gene *chvI* and a conserved hypothetical protein gene. The derivative protein of the gene, a PCK enzyme, had high degree of homology with the known ATP-dependent PCKs and all the putative functional regions of the enzyme were conserved. Phylogeny of the *pckA* gene placed the strain in a cluster with other known members of Rhizobiales. The *pckA* gene was functional in *R. pusense* NRCPB10 and was induced on glucogenic substrates indicating that the regulation mechanism of the gene was also functional. Non-infectivity of *R. pusense* NRCPB10 was not, thus, due to a deficiency in *pckA* gene activity but for some other factor which was not clear in this study.
